# Book Reviews

**Published:** 1901-06

**Authors:** 


### BOOK REVIEWS, PAMPHLETS, EXCHANGES.

### BOOK REVIEWS.

[Contributions solicited for review.]

ATLAS AND EPITOME OF OPHTHALMOSCOPIC DIAGNOSIS. By Professor Dr. O. Haab, Director of the Eye Clinic in Zurich. From the Third Revised and Enlarged German Edition. Edited by Geo. E. de Schweinitz, Professor of Ophthalmology, Jefferson Medical College, Philadelphia. With 152 colored lithographic illustrations and 85 pages of text. Philadelphia and London: W. B. Saunders & Co., 1901. Price, $3.00 net.

The great value of Professor Haab's Atlas of Ophthalmoscopy and Ophthalmoscopic Diagnosis has been fully established and entirely justifies an English translation of his latest edition. Not only is the student made acquainted with carefully prepared ophthalmoscopic drawings done into well executed lithographs of the most important founders changes, but in many instances, plates of the microscopic lesions are added. The whole furnishes a manual of the greatest possible service, not only to the beginner in ophthalmic work, but to one who has already far advanced and desires to compare the observations of his own service with those of the rich clinic from which Professor Haab has gathered his plates.

---

A TEXT-BOOK OF THE PRACTICE OF MEDICINE. By Dr. Herman Eichhorst, Professor of Special Pathology and Therapeutics and Director of the Medical Clinic in the University of Zurich. Translated and edited by Augustus A. Eshner, M.D., Professor of Clinical Medicine in the Philadelphia Polyclinic. Two octavo volumes of over 600 pages each; over 150 illustrations. Philadelphia and London: W. B. Saunders & Co., 1901. Price per set: Cloth, $6.00 net.

The Germans lead the world in internal medicine, and among all German clinicians no name is more renowned than that of the author of this work. Dr. Eichhorst stands to-day among the most eminent authorities of the world, and his Text-Book of the Practice of Medicine is probably the most valuable work of its size on the subject. The book is a new one, but on its publication it sprang into immediate popularity and is now one of the leading text-books in Germany. It is practically a condensed edition of the author's great work on Special Pathology and Therapeutics, and it forms not only an ideal text-book for students, but a practical guide of unusual value to the practising physician. As the essential aim of the physician will always be the cure of disease, the fullest and most careful consideration has been given to treatment.

---

PRINCIPLES OF SURGERY. By N. Senn, M.D., Ph.D., LL.D., Professor of Surgery in Rush Medical College in Affiliation with the University of Chicago; Professorial Lecturer on Military Surgery in the University of Chicago; Attending Surgeon to the Presbyterian Hospital; Surgeon-in-Chief to St. Joseph's Hospital; Surgeon-General of Illinois; late Lieutenant-Colonel of United States Volunteers and Chief of the Operating-staff with the Army in the field during the Spanish-American War. Third Edition. Thoroughly Revised with 230 Wood-engravings, Half-tones and Colored Illustrations. Royal Octavo. Pages, xix—700. Extra Cloth, $4.50 net; sheep or half Russia, $5.50 net. Delivered. Philadelphia: F. A. Davis Company, Publishers, 1914–16 Cherry St.

This work has achieved the position of a classic and comment is well-nigh superfluous. The third edition has received such revision as the progress of medicine has made necessary, and there have been some additions to the work. The new chapters are on Degeneration and "Blastomycetic Dermatitis." New illustrations have also been added. The book is well bound and well printed.

---

THE ACUTE CONTAGIOUS DISEASES OF CHILDHOOD. By Marcus P. Hatfield, A.M., M.D., Professor Emeritus of Diseases of Children, Northwestern University Medical School; Professor of Diseases of Children, Chicago Clinical School; Attending Physician Wesley Hospital. Pages, 142. Price, $1.00 net. G. P. Engelhard & Co., 358–362 Dearborn St., Chicago, 1901.

A clear knowledge of the contagious diseases of childhood is one of the essential requisites of a safe doctor, and a perusal of this book would be desirable to obtain the necessary information. The ground is quite well covered ; the descriptions are lucid, the recommendations for treatment excellent. The work can be commended.

---

ATLAS AND EPITOME OF LABOR AND OPERATIVE OBSTETRICS. By Dr. O. Shaeffer of Heidelberg. From the Fifth Revised German Edition. Edited by J. Clifton Edgar, M.D., Professor of Obstetrics and Clinical Midwifery, Cornell University Medical School. With 14 lithographic plates, in colors, and 139 other illustrations. Philadelphia and London: W. B. Saunders & Co., 1901. Cloth, $2.00 net.

There is no branch of medicine or surgery that is so difficult to demonstrate as that of midwifery; hence any positive aid, such as this Atlas furnishes, is to be hailed with satisfaction. The author has added to the multitude of obstetric subjects already shown by illustration, many accurate representations of manipulations and conditions never before clearly shown. As a guide in the perusal of text-books and as a volume of ready reference, this book will prove invaluable.

---

ATLAS AND EPITOME OF THE NERVOUS SYSTEM AND ITS DISEASES. By Professor Dr. Chr. Jakob, of Erlangen. From the Second Revised German Edition. Edited by Edward D. Fisher, M.D., Professor of Diseases of the Nervous System, University and Bellevue Medical College, New York. With 83 plates and copious text. Philadelphia and London: W. B. Saunders & Co., 1901. Cloth, $3.50 net.

In this Atlas the author has portrayed an instructive section of medicine which is usually extremely difficult of mastery by students and practitioners. This work will be of great value to the physician. The matter is divided into Anatomy, Pathology, and Description of Diseases of the Nervous System. The plates illustrate these divisions most completely. There is probably no work in existence in which so much is compressed within so small a space. The book is comprehensive and practical.

ATLAS AND EPITOME OF OBSTETRIC DIAGNOSIS AND TREATMENT. By Dr. O. Shaeffer, of Heidelberg. From the Second Revised German Edition. Edited by J. Clifton Edgar, M.D., Professor of Obstetrics and Clinical Midwifery, Cornell University Medical School. With 122 colored figures on 56 plates, 38 other illustrations, and 317 pages of text. Philadelphia and London: W. B. Saunders & Co., 1901. Cloth, $3 net.

This book treats particularly of obstetric operations, and besides the wealth of beautiful lithographic illustrations, contains an extensive text of great value. The symptomatology and diagnosis are discussed with all necessary fullness, and the indications for treatment are definite and complete. In this new edition both text and illustrations have been subjected to a thorough revision. Most of the colored plates are new, and illustate the modern improvements in technique as well as a vast amount of new clinical material.

---

A SYLLABUS OF NEW REMEDIES AND THERAPEUTIC MEASURES; With Chemistry, Physical Appearance and Therapeutic Application. By J. W. Wainwright, M.D., Member of the American Medical Association; New York State Medical Association, United States Pharmacopœial Convention, 1900; American Chemical Society, etc. Pages 229. Price, $1 net. G. P. Engelhard & Co., 358–362 Dearborn St., Chicago, 1901.

This work will be found convenient, as it contains the account of many of the drugs which have not as yet been noted by standard text-books, but which are in frequent use and demand. Such are for example, orthoform, norophen, ichthalbin, geosote and the like. Whether they are all of them worthy of mention is a question for the future. The book is well printed and neat in appearance.

---

HARE'S SYSTEM OF PRACTICAL THERAPEUTICS. VOL. III. Lea Bros. & Co. New York and Philadelphia, 1901.

The third volume is devoted to surgery and surgical diseases. The list of contributors includes the names of C. L. Leonard, C. H. Frazer, G. R. Fowler, J. M. Mathews, W. T. Belfield, E. E. Montgomery, H. R. Wharton, Fletcher Ingalls and others. The volume completes the system which is a notable addition to medical literature.

